# Nuts and berries from agroforestry systems in temperate regions can form the foundation for a healthier human diet and improved outcomes from diet-related diseases

**DOI:** 10.1007/s10457-023-00858-8

**Published:** 2023-06-08

**Authors:** Sarah Taylor Lovell, Kiruba Krishnaswamy, Chung-Ho Lin, Nicholas Meier, Ronald S. Revord, Andrew L. Thomas

**Affiliations:** 1 Center for Agroforestry, University of Missouri, Columbia, MO USA; 2 School of Natural Resources, University of Missouri, Columbia, MO USA; 3 Biomedical, Biological and Chemical Engineering, University of Missouri, Columbia, MO USA; 4 Division of Plant Sciences and Technology, Southwest Research, Extension, and Education Center, University of Missouri, Mt. Vernon, MO USA

**Keywords:** Multifunctional agriculture, Tree crops, Biofortification, Tree nuts, Berry crops

## Abstract

Agroforestry is a specific type of agroecosystem that includes trees and shrubs with the potential to yield nutrient-rich products that contribute to human health. This paper reviews the literature on the human health benefits of tree nut and berry species commonly associated with agroforestry systems of the United States, considering their potential for preventing certain diet-related diseases. Emphasis is placed on those diseases that are most closely associated with poor outcomes from COVID-19, as they are indicators of confounding health prognoses. Results indicate that tree nuts reduce the risk of coronary heart disease, and walnuts (*Juglans* species) are particularly effective because of their unique fatty acid profile. Berries that are grown on shrubs have the potential to contribute to mitigation of hypertension, prevention of Type II diabetes, and reduced risk of cardiovascular disease. To optimize human health benefits, plant breeding programs can focus on the traits that enhance the naturally-occurring phytochemicals, through biofortification. Value-added processing techniques should be selected and employed to preserve the phytonutrients, so they are maintained through the point of consumption. Agroforestry systems can offer valuable human health outcomes for common diet-related diseases, in addition to providing many environmental benefits, particularly if they are purposefully designed with that goal in mind. The food system policies in the U.S. might be reoriented to prioritize these food production systems based on the health benefits.

## Introduction

Contemporary food systems have not been designed to offer positive health outcomes as a primary function, despite the fact that the human population faces serious health issues including obesity, metabolic disorder, and micronutrient deficiencies (Garnett 2016; Zhu et al. 2007). Diet-related illnesses have become the primary driver of decreased life expectancy and low quality of life (Gordon et al. 2017). The availability of inexpensive foods that are highly processed and nutritionally-poor corresponds with the incidence of these chronic diseases, and the impacts are often greatest for individuals and communities at low socioeconomic levels (Seligman and Schillinger 2010). While many of our current medical responses to chronic diseases target treatment as opposed to prevention, opportunities exist to integrate plant-based foods and natural medicinal products for a more holistic approach (Martin and Li 2017). Plants hold tremendous genetic diversity contributing to broad arrays of phytochemicals, and various species are adapted and potentially accessible at many locations and scales. Many diverse and lesser-known food and medicinal crops can be grown locally in homegardens, community gardens, food forests, and commercial settings, often without competing for traditional agricultural land (Lovell 2010).

An example of the immense opportunities with locally grown, nutrient-dense plant-based food products and their connection with dietary health was demonstrated following the outbreak of the novel coronavirus, COVID-19. This disease was discovered in late 2019 and rapidly spread across the globe throughout 2020 and 2021. Soon after the outbreak began, it became clear that disease severity was greater among individuals with certain underlying conditions, or “comorbidities”. The conditions shown to have the highest correlations with severe COVID-19 disease outcomes were hypertension (21% of patients), diabetes (9.7%), and cardiovascular disease (8.4%) (Yang et al. 2020). COVID-19 patients who had one or more of these conditions experienced a greater likelihood of developing acute respiratory distress syndrome (Wu et al. 2020), greater admission to Intensive Care Units (Wang et al. 2020), and higher mortality rate (Bansal 2020; Wu et al. 2020). A study of patients in New York City found that obesity was also an important comorbidity, and second only to hypertension as the common factor (Richardson et al. 2020). Hypertension, obesity, diabetes, and cardiovascular disease are each related to the human diet and can typically be improved by changes in eating behaviors to increase intake of certain foods, and decrease others.

**Fig. 1:**
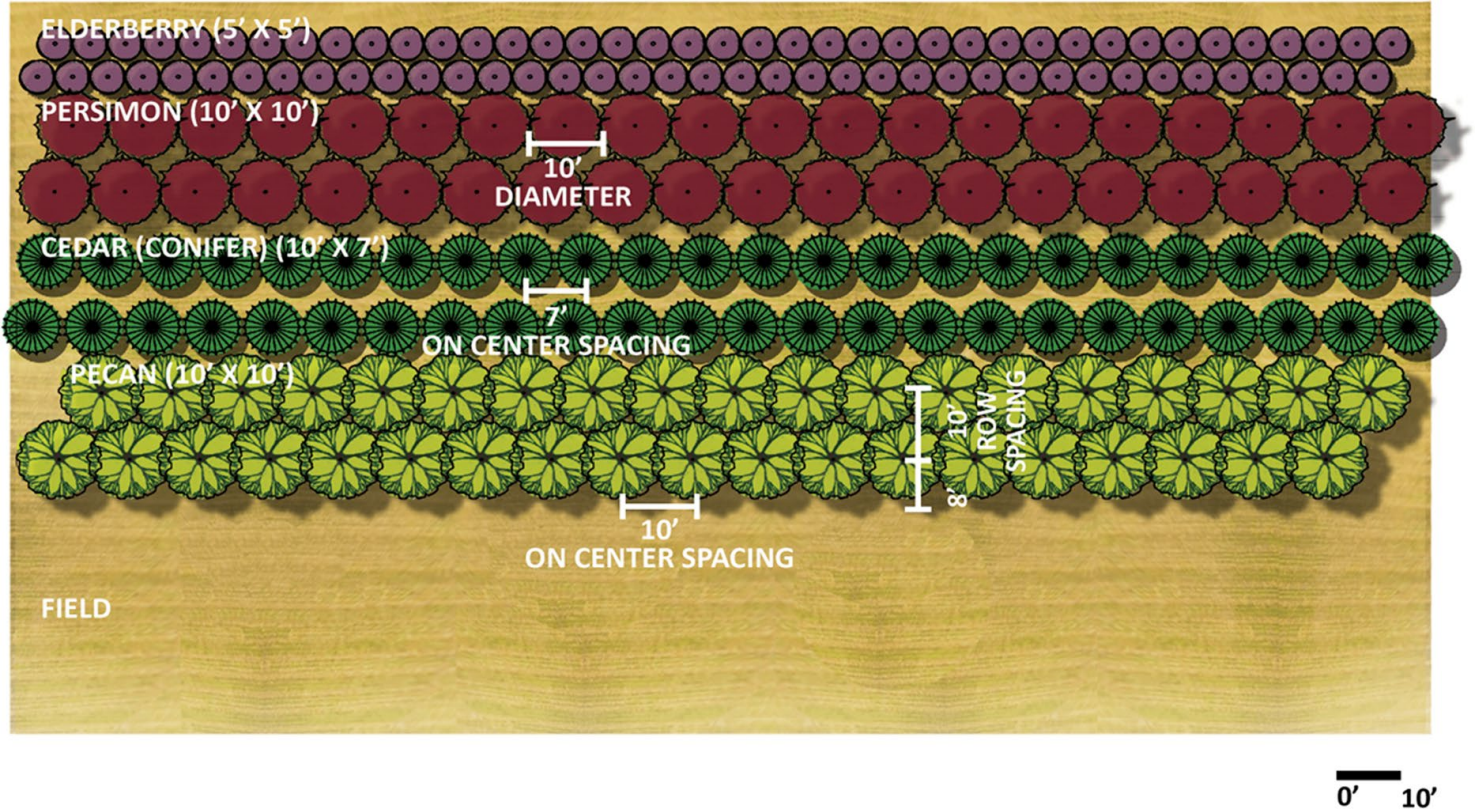
Example of an agroforestry windbreak that is designed to yield healthy food including pecans, persimmon, and elderberry. For optimal production, the pecans trees would need to be thinned to a lower density as they grew larger. The establishment spacing shown here would optimize the environmental benefits (i.e., provide the protection from wind) in early years. Image by Isaac Palomo and Raelin Kronenberg, with permission

Plant-based foods such as fruits, nuts, and leafy greens are among the most important components of a healthy diet that has the potential to reduce these underlying conditions (Slimko and Mensah 2010). Many of these foods can be grown in production systems that also provide environmental benefits. Agroforestry, the integration of trees and shrubs with crops or livestock, can supply healthy food for humans while supporting a healthy environment. Figure 1 depicts an example of one such system. Agroforestry is not a new concept; in fact, many of the agroforestry activities that we encourage today are rooted in practices developed by Indigenous peoples around the world who relied on their deep ecological knowledge to ethically gather and cultivate nuts, fruits, and understory plants for food and medicinal purposes.

In this paper, we explore the potential for a set of plant products associated with agroforestry systems to contribute to preventing or reducing common diet-related diseases. The diseases included here were identified as pre-existing conditions most closely associated with poor outcomes from COVID-19, but they are also connected with other illnesses. In terms of the plant products, we emphasize those that could be included in a temperate region agroforestry system with tree nuts and berry shrubs as the primary components. A very wide range of shade-tolerant herbaceous plants (food crops and medicinal plants) could also be included in the understory, but the review of those alternatives is beyond the scope of this paper. We then consider strategies for developing breeding programs to select for beneficial natural phytochemicals, as well as opportunities for value-added processing to preserve the structure and nutritional values of the products over time in storage.

## Methods

This review is intended as a scientific inquiry to provide a synthesis of interrelated, complex topics encompassing some specific edible plant products along with their impacts on common diet-related diseases that are also known as comorbidities of COVID-19. Databases and search engines used in data collection included Web of Science, PubMed, and Google Scholar during the period of April through July, 2020 and again in June 2022 for newly published relevant articles. After initial assessment of published articles, 71 studies were determined to have content relevant to health benefits of tree nut and berry crops that could be grown in an agroforestry system of the temperate region of the US. Where systematic reviews and meta-analysis studies were available on specific food/disease combinations, those were used to form the basis of general understandings on the topic, rather than compiling the comprehensive literature across all relevant topics. We used snowballing to identify additional sources based on the reference lists of initially identified sources to fill in details specific to the narrative. The details of the most relevant papers, including authors and publication dates, are listed in Tables 1 and 2 of the Results and Discussion section. We do not claim that our review of literature is exhaustive but instead intend for this work to provide a basis for agroforestry system design and an inspiration for future research.

**Table 1:** Summary of key findings on the relationships between consumption of nuts and the health conditions known as comorbidities to COVID-19

Health condition	Crop species	Findings	Reference
Hypertension	Any nut species found in search	Meta-analysis found consumption of nuts reduced risk by 8%	Guo et al. (2015)
	Any nut species excluding peanut	Risk lowered in individuals who were lean, but not in overweight	Djousse et al. (2009)
	Walnuts, almonds, hazelnuts, peanuts	No significant relationship with nut consumption	Martinez-Lapiscina et al. (2010)
Obesity	Any nut species including peanuts	Systemic review, found 4 of 6 studies showed inverse association with nut consumption	Eslami et al. (2019)
	Tree nuts compared with peanuts	Those consuming nuts had slightly lower risk; results similar for tree nuts versus peanuts	Bes-Rastrollo et al. (2009)
	Any nut species including peanuts	Risk reduced 5% for those who consume nuts	Freisling et al. (2018)
	Walnuts, almonds, hazelnuts, peanuts	Consuming nuts 2x/week had slightly lower risk of weight gain	Bes-Rastrollo et al. (2007)
Type II diabetes	Any nut species including peanuts	Meta-analysis indicating no significant response	Guo et al. (2015)
	Tree nuts	Nut consumption resulted in negligible increased risk	Parker et al. (2003)
Coronary heart disease (CHD)	Pooled across all nut species	10% reduced risk for every one serving per week	Weng et al. (2016)
	Pooled across all nut species	Meta-analysis showing 24–30% reduced CHD morality	Kim et al. (2019)
	Walnuts (any Juglans species)	Meta-analysis showing reduced cholesterol with walnuts in diet	Guasch-Ferre et al (2018)
	Black walnuts (*Junglans nigra*)	High concentrations of phytosterols that lower cholesterol	Ostlund (2007); Vu et al. (2019)
	Tree nuts	Found decreases in blood lipids in the order: pistachio > walnut > other tree nuts	Liu et al. (2020)

Peanut (*Arachis hypogaea*), an herbaceous plant typically grown as a commodity crop and not in agroforestry systems, is included along with tree nuts in some studies and not in others. More research is needed to distinguish between the benefits of those different categories

**Table 2:** Summary of key findings on the relationships between consumption of shrub berries and the health conditions known as comorbidities to COVID-19

Health condition	Crop species	Findings	Reference
Hypertension	Blueberry (freeze-dried powder)	Consumption reduced blood pressure compared with placebo	Johnson et al. (2015)
	Red raspberries (frozen)	Consumption resulted in no significant response	Franck et al. (2020)
	Berries in general	Bioactive compounds in berries mitigate hypertension, and specific mechanisms are identified	Yousefi (2021)
Obesity	Berries in general	In mice/rats, results were mixed; concluded more work needed	Tsuda (2016)
Type II diabetes	Berries, many species	Review of literature provides evidence of disease prevention and reduced complications	Calvano et al. (2019)
	Many species, with emphasis on Vaccinium sp.	Review of literature indicates berries help in preventing or managing T2D	Hameed et al. (2020)
Cardiovascular disease (CVD)	Cranberries, strawberries, blueberries, others	Meta-analysis found 2/3 studies with improvements in CHD markers, remaining no change	Heneghan et al (2018)
	Many species	Meta-analysis of 45 studies found reduced number of risk factors for CVD	Luis (2018)

## Results and discussion

Nut trees and shrub berries are gaining popularity as components of temperate agroforestry plantings for sustainable food production. The findings discussed in this section offer evidence that these components can contribute to a healthy diet and improved outcomes from diet-related illnesses. Because a large number of species were included in the review, it was not feasible to provide detailed biological descriptions, taxonomic information, or ecological distributions for each of them. That information can be found through other sources (c.f., Flora of North America Editorial Committee 2023 [REFERENCE: Flora of North America Editorial Committee, 2023. Flora of North America, North of Mexico. Volumes 1-30. New York (NY): Oxford University Press]; Mabberley 2017). Similarly, it is beyond the scope of this paper to include the phytochemical composition (e.g., secondary metabolites) for each species and disease combination. The list of papers in the tables and those cited throughout the paper can be referenced for such detailed information.

### Nuts

Nut trees can be key components of agroforestry systems, and many regions have native species that can be readily included. Nut trees are commonly planted in the tree rows of alley cropping systems (a common agroforestry practice) for commercial orchard production, but they also work well in the upper canopy layer of other agroforestry practices such as riparian forest buffers, windbreaks, and silvopasture where the nuts can be harvested from the ground. Nut-producing trees can take years to become productive, but other crops can be interplanted with a specific goal of obtaining economic returns in the interim (Lovell et al. 2017).

The human health benefits of tree nuts are attributed to nutrient profiles that include high levels of vegetable protein, unsaturated fatty acids (beneficial fats), dietary fiber, vitamins, minerals, and other bioactive compounds (Ros 2010). Worldwide, the most widely consumed tree nuts in 2018 were (in order of consumption): almond (*Prunus amygdalis*), walnut (*Juglans regia*), cashew (*Anacardium occidentale*), pistachio (*Pistachia vera*), and hazelnut (*Corylus avellana*) (Statista 2019). Consumption of tree nuts has been associated with health benefits that include the specific underlying conditions known to impact outcomes of COVID-19: hypertension, coronary heart disease, and diabetes. Table 1 summarizes the results for each of these comorbidities.

#### Nuts and hypertension (high blood pressure)

Studies on the relationship between nut consumption and hypertension have mixed results, with some showing a positive health benefit (reduced hypertension) and others showing no significant relationship. Guo et al. (2015) conducted a meta-analysis on health implications of nut consumption that included three hypertension studies. Findings showed that consumption of nuts at greater than two servings per week resulted in an 8% lower risk of hypertension. A study that used US male physicians as subjects found that nut consumption lowered the risk of hypertension in lean individuals but not in those who were overweight (Djousse et al. 2009). On the contrary, a study of graduate students in Spain found no relationship between nut consumption and hypertension (Martinez-Lapiscina et al. 2010), perhaps due to the young age of the subjects. Studies on the DASH diet which includes nuts have demonstrated a decrease in hypertension (Soltani et al. 2020), but these do not disentangle changes in nut consumption from the other dietary alterations. Overall, while some evidence suggests that nut consumption could reduce hypertension, more studies are needed (Casas-Agustench et al. 2011), particularly to distinguish between tree nuts and peanuts. Peanuts grow as an annual herbaceous crop, most often in large-scale commercial production systems, and might therefore be more appropriately classified as a grain.

#### Nuts and obesity

The relationship between nut consumption and obesity is understudied, and some nutrition experts have been concerned that eating these high-fat foods would result in weight gain. Two separate studies, one labeled a “systematic review of the literature” (Eslami et al. 2019) and the other a “meta-analysis” (Li et al. 2018), both narrowed down to a similar, very small set of studies that met the strict eligibility criteria including the requirement to be a “cohort study”. The most comprehensive study that included both men and women ages 25–70 years old from European countries (n = 373,293) found that those who consumed nuts had 5% lower risk of becoming overweight or obese (Freisling et al. 2018). A study of 51,188 women aged 20–45 found that those consuming nuts at least two times per week had a slightly lower risk of obesity, and there was no difference between tree nut or peanut consumption (Bes-Rastrollo et al. 2009). In a cohort of 937 adult men and woman in the Mediterranean region, those who consumed nuts had a lower risk of weight gain, but the association with obesity was not significant (Bes-Rastrollo et al. 2007). While much more research is needed, the trends suggest that nut consumption does not increase the likelihood of obesity, and for some groups it reduces the risk.

#### Nuts and Type II diabetes

A 2015 meta-analysis on the health effects of nut consumption (including peanuts) revealed six relevant cohort studies on Type II diabetes (T2D). The summary of results from these studies indicated no significant response from consumption of nuts at greater than two servings per week (Guo et al. 2015). For those studies focused on tree nuts, one found a range of negligible to increased risk of T2D with nut consumption (Parker et al. 2003), but most found no significant relationship (Kochar et al. 2010; Montonen et al. 2005; Pan et al. 2013). Another meta-analysis found similar results—no significant association between nut consumption and T2D (Wu et al. 2015). More studies are needed, but current evidence suggests that consuming nuts does not increase or reduce the risk of T2D.

#### Nuts and coronary heart disease

Tree nuts have been shown to provide the specific health benefits of lowering the risk of coronary heart disease (CHD), and this outcome is reflected in the intermediate biomarkers such as blood pressure and blood cholesterol (Ros 2010). A meta-analysis of ten articles that include a total of 14 studies found significantly reduced risk of CHD in high versus low consumption of nuts, with a linear dose–response relationship indicating 10% reduced risk for every one serving per week (Weng et al. 2016). A meta-analysis found that on average, nut consumption decreased CHD mortality by 24–30%, likely due to the lowering of the levels of fasting glucose, total cholesterol, and low-density lipoprotein (LDL) cholesterol (Kim et al. 2019).

Walnuts (*Juglans* spp.) are particularly beneficial for heart health due to their unique fatty acid composition relative to other tree nuts. They contain primarily n-6 and n-3 polyunsaturated fatty acids, linoleic and α-linoleic acid, compared with monounsaturated fatty acids most common in their counterparts including pecans (*Carya illinoinensis*), almonds, and hazelnuts (Mukuddem-Petersen et al. 2005). The fatty acid composition in walnuts appears to have a notable benefit in reducing high-density lipoprotein (HDL-C) (Mukuddem-Petersen et al. 2005). In a meta-analysis of 26 clinical trials that included intervention studies in which walnuts were added to the diet, Guasch-Ferre et al. (2018) found that the reductions in blood lipids (cholesterol) were greatest when walnuts were added to diets of individuals in Western countries (Guasch-Ferre et al. 2018).

### Shrub berries

In agroforestry systems, berry shrubs can be planted between the rows of trees, typically coming into production years before the nut trees are yielding heavily. Some species such as black currant (*Ribes nigrum*) can maintain productivity even when the overstory trees are large enough to compete for light (Wolske et al. 2021). In addition to focusing on shrub species with the greatest potential impacts on chronic diseases, characteristics such as productivity, shade tolerance, adaptability to the area, and low maintenance requirements should be considered when introducing berry shrubs into an agroforestry system.

Numerous scientific studies underscore the roles of dark-colored berry fruits in mitigating serious acute health conditions, but also in addressing some of the underlying causes of chronic degenerative diseases such as obesity, cardiovascular disease, and diabetes. Berry crops such as blackberry and raspberry (*Rubus* spp.), currant and gooseberry (*Ribes* spp.), elderberry (*Sambucus* spp.), aronia berry (*Aronia* spp.), and blueberry (*Vaccinium* spp.) can all play significant roles in promoting and maintaining human health. Some of these berry crops, such as blackberry and blueberry, have well-established production and marketing value chains throughout much of North America, whereas lesser-known crops such as aronia and elderberry are rapidly emerging as their health-providing benefits are becoming well-known (c.f., Schultz 2020). Table 2 summarizes the findings from key studies on diet-related diseases.

#### Berries and hypertension

Fruit intake is generally correlated with lower hypertension (Yu et al. 2018), potentially offsetting the detrimental effect of fat intake (Yuan et al. 2020). Yet, studies specifically with berries are limited (Pinto and Shetty 2010). Studies on rats have shown effective treatment of hypertension using blueberry extract (Turck et al. 2020), aronia berry (*Aronia melanocarpa*) (Cebova et al. 2017), hawthorn (*Crataegus* spp.) (Haydari et al. 2017), and a polyphenol-rich European elderberry (*Sambucus nigra* subsp. *nigra*) extract (Ciocoiu et al. 2016). A limited number of studies have been conducted directly on humans, including one showing that consumption of freeze-dried blueberry powder reduced blood pressure significantly compared with patients receiving a placebo powder (Johnson et al. 2015). Another, however, showed no significant response of consumption of frozen red raspberries on blood pressure (Franck et al. 2020), suggesting the benefits are specific to the species of berry. In a review of the literature, Yousefi et al. (2021) concluded that the bioactive compounds in berries are effective in mitigation of hypertension. The primary mechanisms for efficacy in mitigating hypertension are related to the concentrations of flavonoids, vitamins, minerals, and fibers (Yousefi et al. 2021). Vitamin C consumption can reduce inflammatory markers associated with hypertension and diabetes (Ellulu et al. 2015), suggesting that berries very high in Vitamin C, like black currant (Borges et al. 2010), would be particularly beneficial.

#### Berries and obesity

Berries are rich in anthocyanins which contribute to anti-oxidant properties and may also prevent accumulation of body fat. A review of the literature on this topic found mixed results in obesity prevention, mostly based on studies of mice and rats (Tsuda 2016). Some encouraging results include a study showing that the addition of blueberry extract to the high-fat diet of test mice significantly reduced body weight gain by 20–23% compared with control (high fat diet without blueberry) (Liu et al. 2021). In a study with obese mice that incorporated a European elderberry extract dietary supplement, some obesity related metabolic dysfunctions, including inflammatory response, triglyceride levels, and insulin resistance were improved with elderberry consumption (Farrell et al. 2015a). In a mouse model with hyperlipidemia and HDL (high-density lipoproteins, i.e. "good" cholesterol) dysfunction, mice fed a modest elderberry supplement had reduced fasting serum glucose, improved HDL functions, and significantly lower total cholesterol (Farrell et al. 2015b). A recent study on Saskatoon berry (*Amelanchier alnifolia*) found that the peel of the fruit exhibited a strong ability to inhibit pancreatic lipase enzymes which are known to contribute to obesity (Lachowicz et al. 2019). Given the inconclusive and sometimes contradictory results even within a given study (c.f., Prior et al. 2008), more research is needed to understand the complex relationship between berry consumption and obesity (Tsuda 2016).

#### Berries and Type II diabetes

Evidence suggests that berries can reduce risk and/or complications of T2D, according to recent studies reviewing the literature on the topic (Calvano et al. 2019; Hameed et al. 2020). Several studies have shown that consumption of berries such as blueberry, bilberry, cloudberry, cranberry (all *Vaccinium* spp.), aronia berry, raspberry, lingonberry (*Rubus chamaemorus*), and strawberry (*Fragaria* spp.) can improve insulin responses in healthy adults. Berry consumption has been shown to improve glycemic and lipid profiles, which can be beneficial in diabetes management. While few studies exist specifically on humans, there is evidence that consumption of berries can help in preventing diabetes and reducing complications of the disease (Calvano et al. 2019).

Elderberry and aronia are among the category of “dark berries” that have a wide range of health benefits and very high levels of antioxidants. Badescu et al. (2015) studied the effects of extracts from these two species on diabetic rats with immune system disorders. Both extracts reduced inflammation and modulated immune defenses associated with diabetes (Badescu et al. 2015). *Aronia melanocarpa* extract added to a high-fat diet fed to rats provided a number of promising outcomes related to diabetes: reducing blood glucose, reducing serum insulin levels, improving glucose tolerance, and regulating glucose metabolism enzyme activity (Mu et al. 2020).

#### Berries and cardiovascular disease

A number of systematic reviews have been conducted on the relationship between berry consumption and cardiovascular disease (CVD) or its risk factors (Yang et al. 2019). In a meta-analysis of randomized controlled trials on this topic, Heneghan et al. (2018) found that over two-thirds of studies showed beneficial effects on CVD markers, and the remaining showed no change. Cranberries, strawberries, and blueberries were the most studied berries from that analysis (Heneghan et al. 2018). Luis (2018) published results of a meta-analysis of 45 studies, concluding that intake of berries reduced a number of risk factors for CVD: total cholesterol, LDL-cholesterol, triglycerides, and blood pressure (Luis et al. 2018). A review of the literature by Cassidy (2018) found that increased intake of anthocyanins, which are responsible for the red/blue coloration of many berries, is significantly associated with reduced risk of cardiovascular disease based on disease incidence and favorable changes in biomarkers associated with the risk of CVD (Cassidy 2018).

#### Immunity against coronavirus and influenza

Consumption of fruit from some berry species offers evidence of immunity against viral diseases such as COVID-19, although no studies have been conducted directly on the disease that caused a pandemic, as of the date of this publication. Elderberry in particular has been a source of interest for immunity. We first look at activity against influenza, which has been studied to a much greater extent than coronavirus. In a multi-faceted in vitro study of European elderberry's effect on influenza, Torabian et al. (2019) hypothesized that elderberry's effect is both direct (suppressing infection and cell-to-cell viral transmission), and indirect (modulating release of cytokines) (Torabian et al. 2019). Consistent with this study, other researchers also concluded that elderberry can mitigate the duration, severity, and replication of multiple influenza strains, including H1N1, H3N2, H5N1, and type B strains (Krawitz et al. 2011; Zakay-Rones et al. 1995).

Considering COVID-19 specifically, to our knowledge, human studies have not yet (as of 2021) been published using elderberry or other berry products. In a study by (Chen et al. 2014) on “Infectious Bronchitis Virus”, a pathogenic chicken coronavirus that is not readily controlled by vaccinations, an elderberry extract rendered the virus non-infectious at an early point in infection, and reduced viral titers in cells by four to six orders of magnitude. For a different novel coronavirus, NL63, a stem extract of an Asian elderberry *Sambucus formosana* was effective in reducing the virus yield by inhibiting replication and blocking attachment of the virus, most likely due to the compound caffeic acid (Weng et al. 2019). Future research into the potential for elderberry or other berries to provide immunity against COVID-19 are likely, as we expect the virus will continue to be a health concern in years to come as new variants develop.

In addition to immunity, some phytonutrients in berries may offer benefits in the treatment of COVID-19. Vitamin C, in particular, has been shown to be effective in treating severe respiratory viral infection and may also help with prevention of the infection and better outcomes (Hoang et al. 2020). Berries of sea buckthorn (*Hippophae rhamnoides*) contain approximately 1–4 mg/g of vitamin C (Gutzeit et al. 2008; Sytarova et al. 2020) and substantially higher concentrations are found in leaves (Sytarova et al. 2020). Black currants also offer high concentrations of Vitamin C at around 1.5–2.9 mg/g berries (Mikulic-Petkovsek et al. 2016; Trych et al. 2020).

### Selecting and breeding nut and berry crops for human health

The tree crops covered in this paper contain natural phytochemicals that have not been specifically selected for in breeding programs but instead evolved over time. The phytochemical profiles of plants evolve as a consequence of interactions with their environments, offering adaptive potential to biotic and abiotic stressors. As these stressors are highly variable within and across environments, so are secondary metabolite profiles (Wink 2003). This pool of metabolic diversity, while complex, offers great opportunity to exploit variation beneficial to human health. Over the last century, the central focus of most plant breeding programs has been agronomic traits, largely ignoring flavor and nutrition traits until recent decades. This trend is explained in part by selection emphasizing distinct, observable phenotypes over more complex flavor and health-promoting characteristics (Giovannoni 2018), thus greatly reducing variation for nutrients and secondary metabolites in many cultivated fruits (Klee 2010). Fortunately, wide variation in minerals, vitamins, and phytonutrient profiles is found in broader germplasm collections, landraces, and relatives, and many efforts to improve fruit quality look to these pools for novel genes, as in Karagiannis et al. (2021).

While biofortification can be accomplished through different strategies including genetic engineering (transgenic improvement) and agronomic practices, gains can be made through conventional breeding too. Parental selections containing high concentrations of desired phytonutrients are crossed with desirable agronomic selections over several generations to integrate the quality traits. Initial hybridization is likely to be followed by alternating generations of intercrossing (of unrelated offspring with complementary traits) and backcrossing (intercross selections back to one of the parents). Limitations to the success of conventional breeding are the extent of genetic diversity for the health-promoting traits of interest and the generations/time needed for introgression and new cultivar release (Garg et al. 2018). Modern genetic techniques, however, increase the capacity to reveal the regulatory pathways that produce desired phytonutrient profiles and, in turn, the feasibility of integrating components into clonal selections with agronomically optimized backgrounds. Since fruit and nut crops are clonally propagated, newly bred cultivars can be readily deployed.

### Preserving health-promoting compounds through processing techniques

**Fig. 2:**
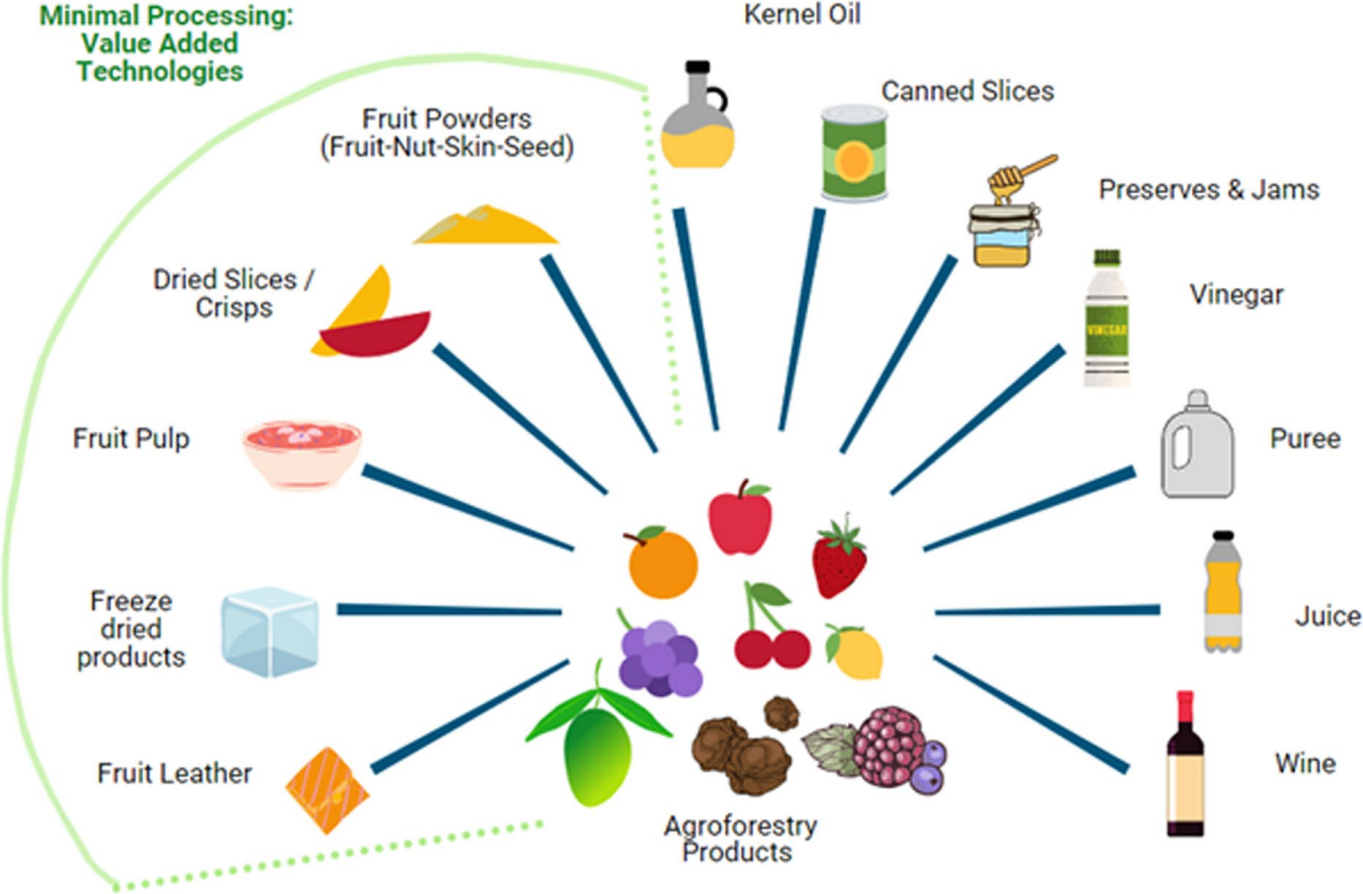
Range of value-added processing technologies, highlighting those that best preserve the phytonutrients from berries and other agroforestry crops

Fruits and vegetables grown in agroforestry systems are highly nutritious and also contain high moisture content and delicate structure, which can lead to food loss and waste. In order to provide the best human health outcomes, modern food processing technologies can be used to preserve the structure and nutritional values of agroforestry food products. Developing integrated post-harvest handling systems will ensure the nutrients are preserved from farm to table and are bioavailable during consumption. Figure 2 shows some of the value-added products that could be developed from agroforestry fruits and nuts.

Berries in particular are rich sources of essential nutrients, so it is important that these phytonutrients are stabilized for greatest benefit in the human diet. They can be consumed as fresh fruits, or pressed, pulped, dried, or powdered via minimal processing. Drying of the fruit can stabilize the essential nutrients without sugar additives, but the process can influence the texture, color, flavor, and phytonutrient content. Solar drying is an ancient technique that can be easily incorporated into small scale processing units (Prakash and Kumar 2013). Hot air drying is one of the most widely used methods, but prolonged drying time could result in nutrient loss and quality degradation (Feng et al. 2012). For example, oven-dried blueberries resulted in a significant loss of vitamin C, total vitamin B, and vitamin B6 compared to freeze-drying (Nemzer et al. 2018). Vacuum drying is an alternative method to oven drying of oxygen- and heat-sensitive products. Vacuum dried aronia berry powder yielded twice the amount of Vitamin C compared to oven drying (Sadowska et al. 2019). Spray drying is a commonly used technique to encapsulate bioactive and therapeutic compounds (Rezvankhah et al. 2020). With freeze drying, also called lyophilization, (Sablani et al. 2011), berries are frozen first before they undergo primary drying with high vacuum conditions then secondary drying to remove unbound moisture. Among freeze-drying, microwave-vacuum drying, hot air drying, and combined hot air-microwave drying techniques, freeze drying was found to be the most effective technique in preserving the total phenolics, anthocyanins, and antioxidant activity (Krishnaswamy et al. 2013; Samoticha et al. 2016). Freeze drying, however, does entail a high cost of operation, high energy use, and a longer drying time. Although the technique is considered to be expensive, it is becoming a more common preservation method due to the superior product quality. For agroforestry products such as tree nuts and berries, value-added processing methods should focus on goals of reducing costs while efficiently preserving health-promoting compounds.

## Conclusions

Agroforestry has been promoted internationally for its broad environmental benefits. This paper explores the potential for agroforestry in the United States to contribute to improved human health. Phytochemicals found in plant products might contribute to the prevention of a number of diet-related diseases, including those that are most closely associated with poor outcomes from COVID-19 and other diseases. Nut-producing trees can make up the overstory of an agroforestry system. As a food product, the greatest health benefits of tree nuts are in the reduced risk of coronary heart disease and related heart-health factors. Berries can be grown on shrubs as a lower layer of a multi-layered agroforestry system. The health benefits of berries have been widely documented and include: mitigation of hypertension, prevention of Type II diabetes, and reduced risk of cardiovascular disease. Furthermore, some berry species may provide immunity or treatment against certain viral diseases. As we look to a future that integrates human health with ecosystem health and sustainable food production, agroforestry has much to offer. By further emphasizing the human health dimensions, new opportunities for broad support may be found through policy and market initiatives. A reorientation of our food system policy is needed to prioritize systems based on the benefits they provide to the broader public.

## Data Availability

Not applicable.
